# SBRT for early-stage glottic larynx cancer—Initial clinical outcomes from a phase I clinical trial

**DOI:** 10.1371/journal.pone.0172055

**Published:** 2017-03-02

**Authors:** David L. Schwartz, Alan Sosa, Stephen G. Chun, Chiuxiong Ding, Xian-Jin Xie, Lucien A. Nedzi, Robert D. Timmerman, Baran D. Sumer

**Affiliations:** 1 Department of Radiation Oncology, University of Texas Southwestern Medical Center, Dallas, Texas, United States of America; 2 Department of Otolaryngology, Head and Neck Surgery, University of Texas Southwestern Medical Center, Dallas, Texas, United States of America; University of Pittsburgh School of Medicine, UNITED STATES

## Abstract

**Purpose:**

To confirm safety and feasibility of hypofractionated SBRT for early-stage glottic laryngeal cancer.

**Methods:**

Twenty consecutive patients with cTis-T2N0M0 carcinoma of glottic larynx were enrolled. Patients entered dose-fractionation cohorts of incrementally shorter bio-equivalent schedules starting with 50 Gy in 15 fractions (fx), followed by 45 Gy/10 fx and, finally, 42.5 Gy/5 fx. Maximum combined CTV-PTV expansion was limited to 5 mm. Patients were treated on a Model G5 Cyberknife (Accuray, Sunnyvale, CA).

**Results:**

Median follow-up is 13.4 months (range: 5.6–24.6 months), with 12 patients followed for at least one year. Maximum acute toxicity consisted of grade 2 hoarseness and dysphagia. Maximum chronic toxicity was seen in one patient treated with 45 Gy/10 fx who continued to smoke >1 pack/day and ultimately required protective tracheostomy. At 1-year follow-up, estimated local disease free survival for the full cohort was 82%. Overall survival is 100% at last follow-up.

**Conclusions:**

We were able to reduce equipotent total fractions of SBRT from 15 to 5 without exceeding protocol-defined acute/subacute toxicity limits. With limited follow-up, disease control appears comparable to standard treatment. We continue to enroll to the 42.5 Gy/5 fx cohort and follow patients for late toxicity.

**Trial registration:**

ClinicalTrials.gov NCT01984502

## Introduction

Laryngeal cancer is the most common non-cutaneous head and neck cancer in the United States, affecting over 13,500 patients annually [1]. Three-quarters of laryngeal cancers originate from the true vocal cords. These are typically detected at an early stage (carcinoma in situ or cT1-T2 tumors) and are curable by single-modality therapy due to low rates of nodal involvement and distant metastasis [2–4].

According to current National Comprehensive Cancer Network (NCCN) guidelines, voice-preserving treatment options for early-stage glottic cancer include CO_2_ laser excision, hemilaryngectomy, and definitive radiation treatment [5]. The use of laser excision has gained popularity for treatment of Stage Tis or T1 lesions for achieving good voice outcomes [6]. Laser ablation involves removal of gross tumor with minimal, often sub-millimeter, excisional margins. However, anterior commissure involvement and larger T2 lesions are technically difficult to resect and may consequently suffer higher risk for local recurrence and/or vocal disturbance [7]. Open hemilaryngectomy is another option for Stage T2 lesions and involves removal of the involved vocal cord and paraglottic space [2, 6, 8]. Radiation therapy is a historically favored non-operative treatment since it provides equivalent disease control with potentially better functional voice outcomes.

Conventional radiation therapy is typically delivered over 6 weeks to a dose of 62–70 Gy [9]. It is inconvenient to patients and treats a large incidental volume of normal tissue. Due to targeting issues, conventional radiotherapy treats both cords and arytenoids, irrespective of involvement. Larynx function may be impaired by radiation-induced edema at these structures.

Stereotactic body radiation therapy (SBRT) minimizes acute and late radiation toxicities by geometrically sparing organs at risk (OAR), while maintaining high-dose treatment to gross disease. The glottic larynx is a feasible site for SBRT and selective sparing of uninvolved vocal fold and/or arytenoid. CT imaging-based analyses confirm that maximum respiratory vocal cord movement at rest is less than 1.3mm [10]. Accelerated hypofractionated larynx radiation treatment has been studied in Europe, with results comparable to conventional therapy [11,12]. In addition to improved patient convenience and cost savings, enhanced tumor cell kill with accelerated hypofractionation could improve local control rates, particularly for high-risk T2 primaries [13]. Potent doses of SBRT, if attainable, would theoretically denude tumor-involved mucosa, similar to laser excision. In light of the excellent local control and functional outcomes achieved with transoral excision, we hypothesized that recapitulating the tight margins and local aggressiveness of surgery with SBRT would significantly improve the therapeutic ratio of non-operative treatment. In this report, we describe interim clinical outcomes for a prospective phase I dose-searching trial utilizing a Cyberknife-based SBRT platform to formally test this approach.

## Methods

### Patient eligibility

The Institutional Review Board of UT Southwestern Medical Center reviewed and approved this study. Written informed consent was obtained from all participants involved in the study. Patients with histologically proven AJCC stage Tis, T1, or T2 squamous cell carcinoma or squamous cell variants (sarcomatoid, verrucous, basaloid, and papillary subtypes) involving the true vocal cords were eligible for this IRB-approved clinical trial (NCT01984502). Study eligibility included age ≥ 18 years and Eastern Cooperative Oncology Group performance status score ≤ 1. Exclusion criteria included evidence of positive nodal or metastatic disease, previous laryngeal surgery or head and neck radiation therapy, and pregnancy. Participant recruitment, data collection, and follow-up for took place in our clinics beginning in November 2013 and remains ongoing.

### Study design

The starting dose-fractionation level was 50 Gy in 15 fractions, with a goal to accelerate treatment to 45 Gy in 10 fractions at dose level 2, and then 42.5 Gy in 5 fractions at the final dose level. These were hypothesized to be equipotent doses per Universal Survival formalism [14]. Furthermore, the 5-fraction treatment goal was predicted to be tolerable based on the published precedents of 1) treating centrally located lung cancer to 50 Gy in 5 fractions without unacceptable airway damage [15] and 2) hypofractionated SBRT-based re-irradiation of recurrent head and neck cancer [16].

**Fig 1:**
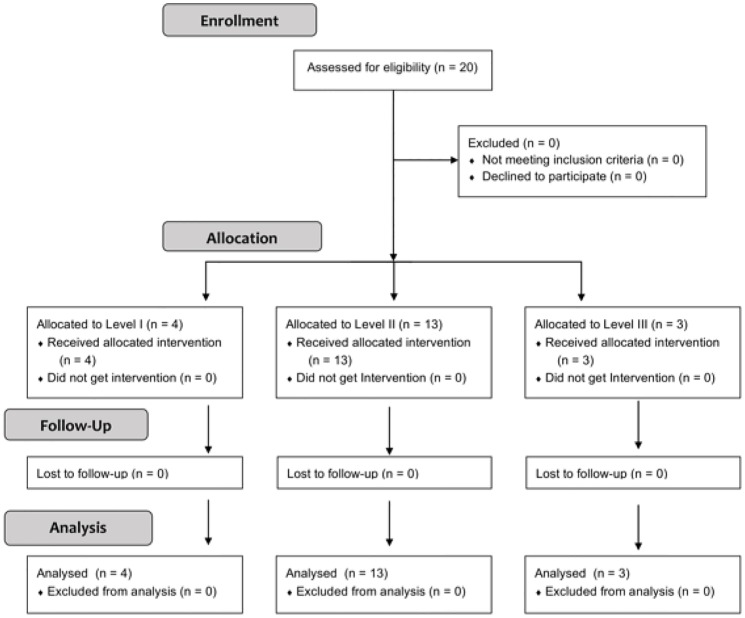
CONSORT diagram.

Fig 1 demonstrates the CONSORT chart for the trial. Toxicity and safety data served as primary outcome measures for this trial. These were assessed at each dose level. To adequately capture all acute toxicity events prior to advancing to a new dose-fractionation cohort, we observed a 90-day delay following completion of treatment of the last patient in a cohort before moving on to the next cohort. DLT was defined as acute or subacute (≤90 days) treatment-related (“possibly”, “probably”, and “definitely”) grade 3 or greater toxicity in the following categories: laryngeal edema, voice, dyspnea, stridor, or cough. No DLTs were observed at the starting dose level in 4 patients. After the 90-day window, the second dose level was opened, with 13 patients subsequently enrolled. Imbalance in cohort size was a result of timing of patient presentation relative to the pre-specified delay between dose-fraction levels. Patients enrolled during delay periods were permitted entry onto the currently open dose-fraction level to minimize accrual interruptions. No DLTs were observed, and the final cohort opened with subsequent enrollment of 3 patients. If >33% of patients enrolled at a given dose level experienced DLT, the maximum tolerated dose would be exceeded. This report summarizes data collected at the time of the first pre-specified interim analysis.

### SBRT technique and dose

Patients underwent a 4-D CT-scan for motion corrected treatment planning. A standard 5-point mask (Qfix, Avondale, PA) was used for immobilization. Intravenous contrast was administered at the time of simulation, and the gross tumor volume (GTV) based on endoscopy, CTV (defined below), and internal target volumes (ITV) were contoured. The protocol originally specified placement of three gold anchor fiducials (Naslund Medical, Chicago, IL) along the thyroid cartilage to assist real-time image guidance. This step was discontinued after we observed minimal laryngeal motion (i.e., CTV = ITV) in the initial 12 patients, allowing standard in-room bone landmark-based image guidance. We observed no complications secondary to fiducial placement.

The clinical target volume (CTV) included GTV plus an isotropic 2 mm expansion. For cT2 lesions, the CTV also included the entire ipsilateral vocal cord and the paraglottic space. For bilateral vocal cord involvement, the CTV included bilateral vocal cords, the anterior commissure, and bilateral paraglottic spaces. The arytenoid was included in the CTV in all lesions coming within 2 mm of or involving the ipsilateral arytenoid. The anterior commissure and 2 mm of the adjacent contralateral vocal cord was included in the CTV in all lesions coming within 2 mm of or involving the anterior commissure. The planning target volume (PTV) was defined as the CTV (ITV) plus a 3 mm uniform expansion.

We identified organs at risk (OAR) as bilateral carotid arteries, bilateral arytenoids, skin, thyroid gland, and spinal cord. The carotid arteries were contoured from the level of the hyoid down to the level of the cricoid inferiorly with an avoidance constraint set to Dmax <10% of prescribed dose. The ipsilateral and contralateral arytenoid cartilage and overlying mucosa were separately contoured. In some cases the ipsilateral arytenoid was part of the CTV and treated to full prescription dose. If not, uninvolved arytenoid tissue was prioritized for sparing (Dmax <26.8Gy and V22.6Gy <0.1cc). The skin was contoured based on the external contour of the body with a Dmax constraint of <10% of prescribed dose. The entire thyroid gland was contoured with a Dmax constraint of <50% of prescribed dose. The spinal cord was contoured from 2 cm superior to the hyoid down to 2 cm inferior to the cricoid with a Dmax constraint of <5% of prescribed dose.

SBRT was delivered in our clinic every other day, 3 fractions/week on a Model G5 Cyberknife^®^ (Accuray, Sunnyvale, CA). The protocol specified premedication of patients with 4 mg of dexamethasone 1 hour prior to radiation therapy, starting with the second fractionation cohort.

Subjects were clinically assessed weekly during treatment for toxicity. Additional follow-up visits were scheduled 4 weeks after radiation treatment and at 3-month intervals for the first year, 6-month intervals for the second year, and annually for years 3, 4, 5. These follow-up visits assessed tumor response, toxicity, and functional voice quality via Voice Handicap Index (VHI) and their quality of life via the (EORTC-QLQ)-H&N35 in conjunction with the EORTC QLQ-30. Surveillance CT imaging was performed at the 3, 6, 12, 18, and 24 month follow-up visits.

### Statistics

Disease outcomes were collected from patient clinical records as secondary study measures and estimated by Kaplan-Meier method. Follow-up interval was calculated from the end of treatment to date of last follow-up. Survival curves were generated using JMP Pro 11 (SAS institute, Cary, North Carolina).

## Results

### Patient characteristics

A total of 20 consecutive patients (18 males/2 females) enrolled between November 2013 and July 2015. Baseline patient characteristics are summarized in Table 1. The median age at time of enrollment was 60 years (range: 39–93 years). There were 14 former smokers, 2 never smokers, and 4 who continued smoking after treatment. Stage distribution was 14 (70%) cT1 and 6 (30%) cT2. Mean GTV, CTV, and PTV volumes were 2.0 ± 1.9, 4.0 ± 2.2, and 8.6 ± 5.7 cm^3^, respectively. Median follow-up was 13.4 months (range: 5.6–24.6 months) with 12 patients followed for at least one year. All patients were alive at time of analysis.

**Table 1:** Patient characteristics.

	Level 1 (n = 4)	Level 2 (n = 13)	Level 3 (n = 3)
**SBRT Dose (Gy)**			
Per Fraction	3.33	4.5	8.5
Total	50	45	42.5
**Age (mean ± s.d.)**	58.8 ± 5.6	63.2 ± 11.9	51.7 ± 16.3
**Gender**			
Male	4 (100%)	12 (92.3%)	2 (67%)
Female	0	1 (7.7%)	1 (33%)
**T Stage**			
cT1a	1 (25%)	7 (53.8%)	2 (67%)
cT1b	2 (50%)	2 (15.4%)	0
cT2	1 (25%)	4 (30.8%)	1 (33%)

### Toxicity and safety

Acute and chronic toxicities at each fractionation level are summarized in Table 2. As noted above, protocol-defined dose limiting toxicities have not been observed. All acute/subacute toxicities have been limited to grade 1 or 2. Most late/chronic toxicities experienced in the first fractionation cohort were grade 1 or less. One patient who recurred had a postoperative fistula/wound infection event following salvage laryngectomy, which has resolved. At the second fractionation level, maximum acute/subacute toxicity consisted of grade 2 hoarseness and dysphagia. Late/chronic toxicities were mostly grade 1 or less, but did include one patient with grade 3 dysphagia and grade 4 laryngeal edema developing 5 months after treatment. This patient had continued to smoke >1 pack cigarettes/day during and following treatment, and ultimately required protective tracheostomy. At the third fractionation level, maximum acute/subacute toxicity was grade 1 laryngeal edema and dermatitis; no late/chronic toxicities have been observed.

**Table 2:** Treatment toxicities.

	Level 1 (n = 4)		Level 2 (n = 13)		Level 3 (n = 3)	
	Acute	Chronic	Acute	Chronic	Acute	Chronic
**Grade 1**						
Laryngeal Edema	0	4	4	1	1	0
Dry Mouth	1	1	3	0	0	0
Dermatitis	0	1	2	0	1	0
Dysphagia	2	0	0	0	0	0
Dysguesia	0	0	2	0	0	0
Cough	0	0	1	0	0	0
Hoarseness	1	0	1	0	0	0
**Grade 2**						
Hoarseness	0	1	1	0	0	0
Dysphagia	0	0	1	0	0	0
**Grade 3**						
Dysphagia	0	0	0	1	0	0
**Grade 4**						
Laryngeal Edema	0	0	0	1	0	0

### Disease outcomes

**Fig 2:**
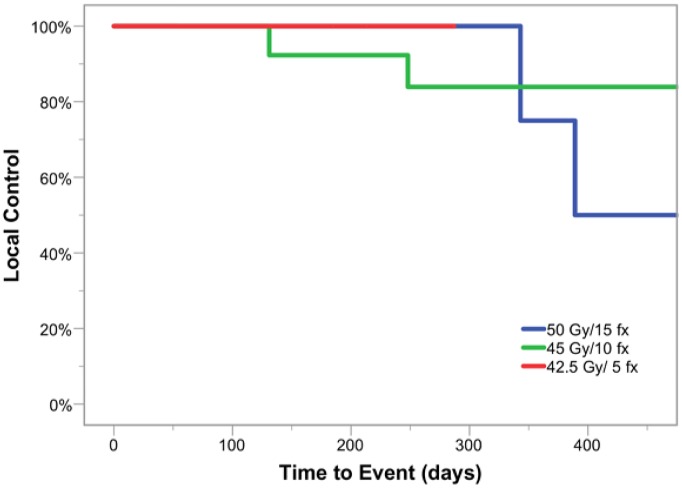
Estimated local recurrence free survival by fractionation cohort.

**Fig 3:**
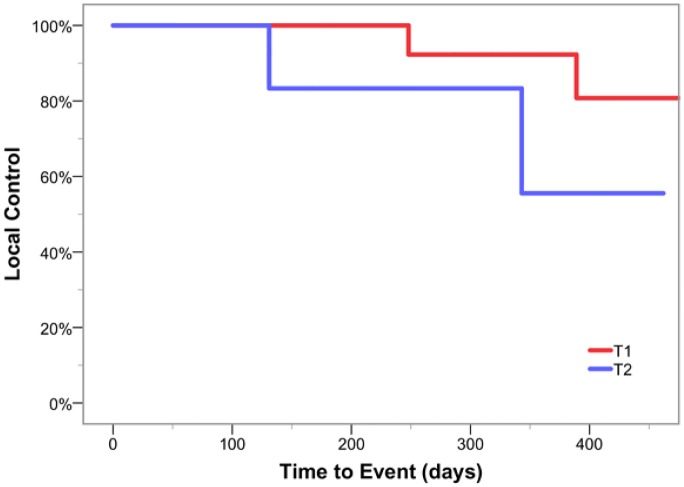
Estimated local recurrence free survival for cT1 and cT2 lesions.

Preliminary disease control measures were collected as secondary outcomes for this phase I study. At 1-year follow-up, estimated local control was 82% and overall survival was 100%. Four patients experienced recurrences within the treated glottic larynx. Local control by fractionation cohort is shown in Fig 2. Two patients (one in fractionation cohort 1, one in cohort 2) with cT1 disease experienced in-field failure and were salvaged via laser resection of persistent in situ or superficially invasive disease in high-dose regions. Two additional patients (one in cohort 1, one in cohort 2) with cT2 primaries with subglottic disease extension experienced failure in high-dose target volumes requiring salvage laryngectomy. Estimated local recurrence free survival curves for cT1 and cT2 lesions are shown in Fig 3. All patients currently remain disease free following salvage surgery at last follow-up.

## Discussion

These initial results suggest that our method of volume-limited SBRT for early-stage glottic laryngeal cancer allows reduction in the total fractions from 15 to 5 without exceeding our protocol-defined toxicity limits. The starting fractionation level used in this study was based on experience from Gowda, et. al. demonstrating safety and efficacy with 50–52.5 Gy in 16 daily fractions for early T1 glottic lesions [11]. We observed a solitary severe toxicity event with 10-fraction treatment in a patient who continued to smoke heavily through treatment. This mirrors the experience of Gowda, *et*. *al*., who reported similar toxicity in a persistent smoker. It is possible that at accelerated fractionation, elevated risk for severe toxicity may be more prevalent in patients who continue to smoke. On the other hand, recent data suggests that the glottic larynx may itself be susceptible to aggressively hypofractionated SBRT, at least in the setting of re-irradiation to a previously treated neck [17]. More conclusive interpretation of this question, as well as overall chronic toxicity following partial larynx SBRT will be critical and awaits ongoing accrual and surveillance.

Local control rates for early glottic laryngeal cancer range between 80–95% for T1 primaries, and 70–80% for T2 lesions [2, 3, 18]. Ultimate control rates exceed 90% with surgical salvage, typically consisting of total laryngectomy [2]. High local control rates should shift focus onto reducing dose to surrounding normal structures since conventional therapy exposes organs-at-risk (contralateral vocal cord, arytenoids, low pharyngeal constrictors) to full dose. In fact, IMRT-based single cord irradiation strategies are currently being investigated [19]. Our initial dosimetric review indicates that our SBRT approach using sophisticated near real-time image guidance improves upon selective IMRT larynx treatment (manuscript submitted).

Our initial clinical results demonstrate an overall local control rate of 82% at 1-year follow-up, comparable to conventional treatment despite considerably smaller target volumes with the SBRT technique. These results are not surprising. With transoral minimally invasive surgery, resection is confined to removal of gross tumor with minimal excision margins. This approach provides excellent cancer control. The two T1 failures in this series were salvaged by larynx-sparing transoral laser excision. Unfavorable T2 tumors may remain at higher risk for local failure and require total laryngectomy for salvage. Further treatment acceleration to 5-fraction SBRT may improve control, similar to improved 3-year local control rates of lung cancer seen with 5-fraction SBRT [20]. Published data suggest that the factor most predictive for local control following radiotherapy is overall treatment time, which is limited to less than 2 weeks with 5-fraction SBRT [13]. It is also important to note that SBRT outcomes will be tightly operator-dependent, mirroring laser excision outcomes. Our SBRT target volume is limited to visible tumor plus a modest margin, similar to laser excision. CyberKnife radiation depends on precise technique with very rapid dose drop-offs. Therefore, technical refinement, standardization, and patterns of disease failure analysis will be critical for successful maturation of this strategy.

The potential cost and convenience savings for patients with hypofractionated treatment are significant. Transoral laser excision of T1 glottic cancers has recently grown in popularity [21, 22], likely due to the relative convenience of laser excision over conventional radiotherapy. The logistical hardships of protracted radiation treatment represent a significant opportunity cost [23]. Hypofractionated SBRT would reduce patient inconvenience and clinic resource utilization, while potentially maintaining favorable voice and tumor control outcomes. Similar to surgery transitioning from open to transoral approaches with limited resection volumes, SBRT offers a promising strategy for reducing treatment volume while maintaining or improving oncologic control.

## Conclusions

We report initial clinical results from a phase I SBRT trial for early-stage glottic cancer. Toxicity profiles appear favorable, without appearance of protocol-defined DLTs. Pilot treatment outcomes appear comparable to standard treatment, albeit with short follow-up. Two T1 tumor recurrences could be salvaged with transoral laser excision. Two larger T2 tumor recurrences required total laryngectomy for salvage. Patients who continue to smoke heavily may be at risk for SBRT—associated toxicity as a late event; however, continued surveillance will be required confirm this interpretation, as well as overall acceptability of chronic toxicity. This phase I trial remains ongoing, with patient accrual continuing onto the 5-fraction treatment arm.

## Supporting information

S1 File UTSW larynx SBRT phase I protocol. (PDF)

S2 File TREND statement checklist. (PDF)
