# Supplementary material for: SBRT for early-stage glottic larynx cancer—Initial clinical outcomes from a phase I clinical trial
Source: PLoS One. 2017 Mar 2;12(3):e0172055. doi: 10.1371/journal.pone.0172055 (PMC5333979; doi:10.1371/journal.pone.0172055)
Supplement: S1 File — (PDF) [file pone.0172055.s001.pdf]

A Phase I CyberKnife Accelerated Hemilarynx Stereotactic Radiotherapy Study for Early-stage Glottic Larynx Cancer

Organizing Institution:
Department of Radiation Oncology
Harold C. Simmons Comprehensive Cancer Center
University of Texas at Southwestern Medical Center

Principal Investigator: Baran Sumer, MD
Department of Otolaryngology
University of Texas at Southwestern Medical Center
5323 Harry Hines Boulevard
Dallas, TX 75390
Phone: (214) 648-2432
Email: Baran.Sumer@UTSouthwestern.edu

Co-Principal Investigator: David Schwartz, MD
Department of Radiation Oncology

Co-Investigator(s):

Ramzi Abdulrahman, MD
Department of Radiation Oncology

Stephen Chun, MD
Department of Radiation Oncology

Chiuxiong Ding, PhD
Department of Medical Physics

Ted Mau, MD, PhD
Department of Otolaryngology

Larry Myers, MD
Department of Otolaryngology

Lucien Nedzi, MD
Department of Radiation Oncology

Peter Roland, MD
Department of Otolaryngology

Robert Timmerman, MD
Department of Radiation Oncology

John Truelson, MD
Department of Otolaryngology

John Yordy, MD, PhD
Department of Radiation Oncology

Xian-Jin Xie, PhD
Biostatistics

Protocol Version	Date
Version 1	10/30/2013
Version 2	01/07/2014
Version 3	06/23/2014

Signature Page

The signature below constitutes the approval of this protocol and the attachments, and provides the necessary assurances that this trial will be conducted according to all stipulations of the protocol, including all statements regarding confidentiality, and according to local legal and regulatory requirements and applicable U.S. federal regulations and ICH guidelines.

Principal Investigator (PI) Name: **Baran Sumer, MD**

PI Signature: ______________________________

Date:_____________________

INDEX

Schema

1.0 Background and Rationale

2.0 Study Objectives

3.0 Subject Eligibility

4.0 Treatment Plan

5.0 Study Procedures

6.0 Data Safety Monitoring Plan

7.0 Data Collection

8.0 Quality of Life

9.0 Quality of Life and Cost Effectiveness

10.0 Statistical Considerations

References

STUDY SCHEMA

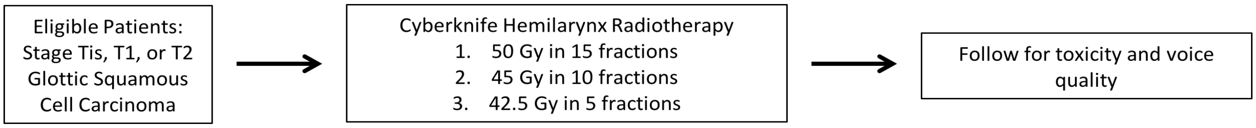

Number of patients = Minimum: 17, Maximum: 48

Phase I

Patients in each dose cohort will all be treated as a single group for fraction reduction. The starting dose will be 3.33 Gy per fraction for 15 fractions (total dose = 50 Gy). Subsequent cohorts of patients will receive a biologically equivalent dose delivered in 5 fewer fractions per step. If significant toxicity is encountered at the starting dose, an increase in fraction step will occur (step -1) to 3.28 Gy per fraction for 16 fractions (total dose 55 Gy).

Step No.	Fractions	Dose per fraction (Gy)	Total Dose (Gy)	No. Patients
-1	16	3.28	52.5	3
0 (starting)	15	3.33	50.0	3-15
1	10	4.50	45.0	7-15
2	5	8.50	42.5	7-15

Eligibility (see Section 3.0 for details)

Patients who satisfy all of the following conditions will be eligible for this study:

- Biopsy proven cT1 and cT2 glottic carcinoma or carcinoma *in situ*
- Willing and capable to provide informed consent
- Signed study specific informed consent form
- Age > 18
- Eastern Cooperative Group (ECOG) Performance Status 0-1
- Appropriate staging studies identifying as AJCC stage 0 (Tis), I, or II true glottic vocal cord laryngeal cancer
- Agreement to use effective contraceptive methods such as condom/diaphragm and spermicidal foam, intrauterine device, or prescription birth control pills
- Patients with a history of non-head/neck invasive malignancies are eligible if they have been disease free for 3 or more years prior to entry into the study

1.0 BACKGROUND AND RATIONALE

1.1 Disease Background

Laryngeal cancer is the most common non-skin head and neck malignancy affecting approximately 13,000 patients annually in the United States.[1] Over 75% of laryngeal cancers involve the true vocal cords or the glottic larynx, with over 90% of these cancers detected in early-stages (defined as carcinoma *in situ* and cT1-T2 tumors) when they are potentially curable by single-modality therapy. Local therapies are highly effective at curing early-stage glottic cancer, as rates of nodal involvement and metastatic disease are less than 5%.[2-4] Treatment outcomes with radiation or surgical approaches for early-stage glottic laryngeal cancer yield local control rates for Tis and T1 tumors greater than 90%, and for T2 tumors 70-80%, and ultimate control rates exceed 90% after surgical salvage.[2,5]

There are multiple voice-preserving local treatment options for early-stage glottic laryngeal cancer in the current National Comprehensive Cancer Network (NCCN) guidelines.[6] These include conventional fractionated radiation therapy, CO_2 laser excision, and hemilaryngectomy. Conventional radiation therapy of the larynx remains the most common treatment for Stage Tis, T1, and T2 glottic tumors typically delivered over 6 weeks to a dose of 62-70 Gy.[7] Although functional voice outcomes from radiation therapy have historically been considered to be superior to surgery, daily radiation treatments over 6 weeks are inconvenient and unnecessarily irradiate large volumes of uninvolved normal tissue. The use of CO_2 laser ablation has gained popularity for the treatment of Stage Tis or T1 lesions, which involves the removal of the gross tumor with a 2-3 mm margin thereby preserving the involved vocal cord and achieving good voice outcomes.[8] Hemilaryngectomy is another voice preservation option particularly for selected Stage T2 lesions where the involved vocal cord and paraglottic space are removed, with additional resection of the ipsilateral arytenoid and/or anterior commissure if involved.[2,8,9] Given the inconvenience of conventional radiation therapy, there is interest in developing hypofractionated stereotactic radiation approaches for early-stage glottic cancer to provide a non-invasive convenient treatment option.

Accelerated hypofractionated irradiation of early glottic cancer has been primarily used in Europe with results similar to conventional radiation therapy.[10,11] Hypofractionated laryngeal irradiation was initially used during World War II due to a shortage of hospital beds. Even in this early experience with primitive radiation techniques, there was no difference in cure rates when reducing radiation therapy from a 5 week course to a 3 week course.[12] Thereafter, the British Institute of Radiology Fractionation study for laryngeal and pharyngeal cancer showed equivalent survival rates and no significant differences in acute or long-term toxicity when comparing a 3-week or 6-week radiation course.[11] In contemporary radiation practice, the Royal Marsden Hospital experience of 200 patients with T1 glottic cancer treated with accelerated radiation therapy to a dose of 50-52.5 Gy in 16 daily fractions, showed outcomes comparable to historical controls with a 93% 5-year local control rate and only a single severe toxicity occurring in a patient who continued to smoke more than 40 cigarettes per day during and after radiation treatment. In addition to patient convenience and cost-savings advantages, hypofractionated radiation therapy has the potential to further improve local control rates, especially as prolonged radiation courses are associated with lower local control and survival rates.[13] A phase III clinical trial in Japan has also shown significantly improved local control in favor of hypofractionation when comparing 56.25 Gy in 25 fractions to conventional 60-66 Gy in 30-33 fractions in early-stage glottis cancer with equivalent minimal toxicity.[14] These lines of evidence suggest that further hypofractionation has multiple potential benefits in early-stage glottic cancer including improved local control, cost savings, and patient convenience.

Given the success rates of local surgical treatments with CO_2 laser ablation and hemilaryngectomy, there has been interest in developing hypofractionated hemilarynx stereotactic body radiotherapy (SBRT) treatment strategies for early-stage glottic cancer. SBRT has the potential to achieve excellent local control rates by delivering ablative tumor doses and to reduce acute and late radiation toxicities by geometrically sparing organs at risk (OAR) with steep radiation dose gradients. During respiratory motion, 4-D CT-scan analyses have shown the maximal movement of the vocal cords to be less than 1.3 mm, providing a feasible target for SBRT.[15] Dosimetric analyses have also shown the sparing significant dose to pertinent organs at risk including the contralateral uninvolved vocal cord, arytenoids, swallowing muscles, strap muscles, carotid arteries, thyroid gland, and laryngeal skeleton using IMRT-based single cord irradiation.[16,17] In particular, dose to the contralateral uninvolved vocal cord could be limited to less than 30 Gy which has the potential to improve functional voice outcomes. Based upon these dosimetric analyses, it was concluded that IMRT could be used to treat early-stage glottic tumors with 1-2 mm margins using ablative stereotactic radiation doses through single cord targeting.[17] Presently, IMRT-based stereotactic hemilarynx irradiation protocols are under development in the Netherlands.[16] Using CyberKnife radiation techniques, our dosimetric analyses suggest potential for even greater normal tissue sparing, and the potential for dose escalation or fraction reduction.

1.2 Study Agent(s) Background and Associated Known Toxicities

‘Stereotactic radiosurgery’ generally refers to a procedure design to treat deep-seated brain tumors or abnormalities, and is commonly performed on a specialized machine, such as the Gamma Knife. This procedure involves immobilizing the patient (cranial halo), affixing a stable 3-D coordinate system (fiducial box and head frame), performing high resolution imaging (CT or MRI), registering the images to the coordinate system using a computer, virtually simulating delivery of very focal and conformal dose profiles of radiation with steep dose gradients toward normal tissue, and finally carrying out the treatment with sub-millimeter accuracy. Typically very high doses of radiation (15-40 Gy) are given in a single treatment with this technique. Any adjacent normal tissues that receive this dose may be significantly damaged, thus the requirement for very conformal treatments with rapid dose fall-off. An alternate strategy has been to divide total radiation dose into two or three fractions, still with fairly large dose per fraction (6-10 Gy), attempting to decrease adjacent normal tissue toxicity. These fractionated techniques are referred to as ‘stereotactic radiotherapy,’ and are carried out with hope that surrounding normal tissue will tolerate the treatment as a result of relatively more successful sublethal damage repair as compared to tumor.

Translation of the stereotactic radiosurgery and radiotherapy concepts to extracranial sites has not been straightforward.[18,19] With brain treatments, the skull serves as an excellent surface to rigidly couple the immobilization frame using stainless steel pins under local anesthesia. Once the skull is immobilized, targets within the skull are likewise immobilized in that there is very little movement of intracranial structures outside of fluid waves around the ventricles. Such is not the case for extracranial sites. Inherent motion, such as the heart beating, lungs expanding and emptying, and bowels churning, results in movement of potential targets. In addition, the external surface anatomy does not have structures amenable to rigid fixation to a frame. In 1994, Lax, et al, from the Karolinska Hospital in Sweden reported on the development and testing of an extracranial frame that incorporated a fiducial stereotactic coordinate system along its side panels.[20] The system used vacuum pillows to make contact with three sides of the patient (maximizing surface area of contact) and correlation of external anatomical reference points on the sternum and calf for immobilization. To decrease respiratory excursion, an abdominal press was employed forcing the patient to perform relatively more chest wall rather than diaphragmatic breathing. A formal verification of reproducibility study was carried out, and

target motion was reduced to within 0.5 cm in the axial plane and 1.0 cm in the caudal/cephalad plane. With this degree of accuracy (compared to 0.05 cm target position accuracy for the Gamma Knife), stereotactic radiosurgery could not be performed; however, they did set up a program treating patients with extracranial stereotactic radiotherapy.

Stereotactic body radiation therapy (SBRT) is a new therapeutic paradigm for treating localized tumors outside of the central nervous system and involves delivering very high doses of focused radiation using unique beam arrangements and special immobilization equipment. As already demonstrated in lung and liver cancers, these treatments offer hope for improved local control of cancers that may translate into gains in survival especially for smaller early stage lesions. SBRT employs daily treatment doses dramatically higher than typical for conventionally fractionated radiation therapy (CFRT). In turn, it is incorrect to assume that SBRT radiobiology is similar to historical CFRT. Indeed, a unique biology of radiation response for very large dose per fraction treatments is being appreciated both in terms of tumor control as well as normal tissue consequences translating into unique clinical outcomes. For example, local control with CFRT in early stage lung cancer is consistently reported below 50% while several series using SBRT show local control around 90%.[21-23]

SBRT has been defined by the American College of Radiology (ACR) and American Society of Therapeutic Radiology and Oncology (ASTRO) to involve the use of very large dose per fraction.[24] Indeed, dose per fraction of 6 Gy minimum would obviously make SBRT very different from even the more abbreviated hypofractionation schemes described above. Typically, only 1-5 fractions are used for SBRT depending on the tolerance of adjacent or intervening normal tissues. Linear structures (like the spinal cord) and tubular structures (like the bowels) are commonly called “serially functioning tissues” akin to series electrical circuits because their function is disrupted if there is a defect anywhere along their pathways.[25,26] It has been shown that serial functioning tissues are less tolerant to SBRT than so-called “parallel functioning tissues” like the peripheral lung and liver. In response, typically more fractions are employed (e.g., five fractions rather than one) when serially functioning tissue cannot be avoided.

Accuray’s Cyberknife system® is a 6 MV linear accelerator mounted on a robotic arm that is unconstrained by the gantry rotations of conventional linear accelerator radiotherapy systems. This allows for delivery of automated non-coplanar treatments and allows for increased conformality and dose gradient. The system also includes image guidance throughout the treatment using several available tracking systems that can track both bony anatomy or fiducial markers. These technologies make the Cyberknife system® ideal for delivering conformal high dose treatment plans while providing continuous image guidance and targeting corrections for quality assurance during treatment. The Cyberknife system® has been used in both intracranial radiosurgery and SBRT treatment of both lung and prostate.[27-29]

1.3 Current Protocol

There are multiple therapeutic options for early-stage glottic larynx cancer with similarly high local control and overall survival rates as mentioned above. However, surgical approaches are invasive and may have worse voice outcomes, while conventional radiation therapy is inconvenient and treats large volumes of uninvolved normal tissue. Using CyberKnife-based SBRT, this protocol will spare adjacent uninvolved normal tissue for the purpose of decreasing the number of fractions required for curative treatment.

As surgical ablation and resection have excellent local control and cure rates for early stage glottis larynx cancer, the “radiosurgery” volumes will emulate volumes of tissue

typically removed or ablated during surgery. Thus, the radiation volume will approximate CO2 laser excision for carcinoma *in situ* and cT1 lesions which typically includes 2 mm of adjacent vocal cord, and the radiation volume for T2 lesions will emulate volumes of tissue typically removed during voice preserving hemilaryngectomy.

Through hypofractionated SBRT with geometric avoidance of uninvolved normal tissue, we aim to increase convenience, decrease toxicity, and improve local control compared to other methods of radiation treatment. As SBRT is a local treatment, eligible patients will be limited to early-stage laryngeal cancer defined as AJCC Tis, T1, and T2 lesions that have less than a 5% risk of nodal or distant metastases at diagnosis.

The feasibility of reducing fractions in early-stage laryngeal cancer has not previously been prospectively studied, and we will therefore conduct a careful phase I bio-equivalent dose fraction reduction study. Patients enrolled at each dose level will undergo routine evaluations to identify potential toxicities as well functional voice analyses. Adequate waiting periods will be used to ensure that fraction reduction does not proceed prior to observing toxicity. The purpose of the study will be either to determine the maximal fraction reduction possible until a dose is reached where a dose-limiting toxicity occurs. The initial dose and fractionation will be 50 Gy in 15 fractions with the goal to reduce number of fractions to a dose of 42.5 Gy in 5 fractions. The rationale for using 5 fractions is that it is tolerated in centrally located lung tumors at a dose of 50 Gy in 5 fractions, and thus, is likely to be tolerated in large calliber airways.[30]

We hypothesize that the larynx has a similar radiation toxicity profile as the central lung airways and can be treated safely using 5 fractions. Potential dose limiting toxicity from this treatment will likely relate to voice hoarseness, stridor, or cough. Strict dosimetric guidelines to reduce the probability of toxicity will be used according to our institutional dosimetric analyses of CyberKnife larynx irradiation of anthropomorphic phantoms.

1.4 Starting dose for Phase I study

Previous experience in Europe suggests that the treatment of early-stage laryngeal cancer with 50-52.5 Gy in 16 fractions is safe and efficacious using conventional radiation techniques.[10,11] Therefore, we hypothesize that with CyberKnife stereotactic radiotherapy techniques, that further fraction reduction is possible. The starting dose for this protocol will be similar to European protocols, and will be 50 Gy in 15 fractions daily. The current protocol will aim to reduce fractions while maintaining excellent local control and minimal toxicity that is seen with conventional radiation techniques.

1.5 Voice Quality and Symptom Assessment

In light of several recent reports describing the voice quality as measured by the Grade-Roughness-Breathiness-Aesthenicity-Strain (GRBAS), Voice Handicap Index (VHI), and various video stroboscopy techniques assessing laser resection and stripping, we will assess voice quality longitudinally as well through the use of VHI assessment[31-35]. Due to inability to objectively evaluate voice through stroboscopy or other computer models, we will assess the voice quality subjectively via the VHI [36]. The voice handicap index is a 30 question assessment evaluating the functional physical and emotional impact that voice handicap has on a patient. It is estimated that this form takes five minutes to complete.

In addition, we will assess swallowing function as measured by the MDADI (MD Anderson Dysphasia Inventory) [37]. This is a 20 question self-administered questionnaire designed to evaluate the impact of dysphasia on the quality of life of patients with head and neck cancer and it is estimated this form takes 5 minutes to complete.

1.6 Health Related Quality of Life

With the establishment of the Patient Centered Outcome Research Institute (PRCORI) funded by the Patient Protection and Affordable Care Act, there is an increase interest in the impact of new treatment technologies on the quality-of-life, outcomes, clinical effectiveness and appropriateness of new technologies. Health-related quality of life (HR-QoL) or patient reported outcomes (PRO) are an important aspect of clinical outcomes. As such, there numerous studies reporting health-related quality of life in the management of early stage glottic carcinoma summarized in a recent systematic review of these studies evaluating radiotherapy and various surgical techniques including trans oral laser microsurgery or stripping [38]. Unfortunately, there have been numerous health-related quality of life forms used to assess patients undergoing treatment for early-stage glottic cancer drawing conclusions between studies difficult. We will utilize the EORTC QLQ 30 and H&N35 which have been used in prior studies evaluating the treatment of early-stage glottic cancer [39-41]. Additionally these forms are already in clinical use in our department as clinical practice.

To aid the ability to assess this technology from a cost-effectiveness standpoint will also assess patient preferences/utility of the treatment through the use of the Euro-QoL EQ 5D utility assessment. See section 1.7 regarding cost-effectiveness analysis.

2.0 STUDY OBJECTIVES

2.1 Primary Objectives

2.1.1 To determine the feasibility of fraction reduction for early-stage laryngeal cancer without exceeding the maximum tolerated dose.

2.2 Secondary Objectives

2.2.2 To determine overall survival at 5 years.

2.2.3 To determine loco-regional control at 5 years as determined by physical exam, visualization of tumor by layngoscopy, and CT-scan of the neck to determine if the primary tumor is controlled. A tissue biopsy or recurrent or persistent disease will be required to be considered a loco-regional failure.

2.2.4 To characterize functional voice quality of patients treated on this protocol.

2.2.5 To characterize the HR-QoL and PRO of patients treated on this protocol.

2.2.6 To determine cost-effectiveness of hypofractionated larynx irradiation.

2.2.7 To determine late toxicity as defined as treatment-related toxicity occurring ≥ 18 months from completion of radiation therapy.

3.0 Subject Eligibility

Eligibility waivers are not permitted. Subjects must meet all of the inclusion and exclusion criteria to be registered to the study. Study treatment may not begin until a subject is registered.

3.1 Inclusion Criteria

3.1.1 Stage Tis, T1, or T2 laryngeal squamous cancer as defined by American Joint Commission on Cancer (AJCC) 2007 staging system
3.1.2 Biopsy proven squamous cell carcinoma histology or squamous cell variants (sarcomatoid, verrucous, basaloid, and papillary subtypes) involving the true vocal cord
3.1.3 Direct laryngoscopy showing no evidence of greater than Stage II true glottic larynx cancer
3.1.4 Chest X-ray or CT-scan showing no evidence of metastatic disease
3.1.5 CT-scan of the neck showing no evidence of nodal involvement
3.1.6 Age ≥ 18 years.
3.1.7 Women of child-bearing potential and men must agree to use adequate contraception (hormonal or barrier method of birth control; abstinence) prior to study entry, for the duration of study participation, and for *90* days following completion of therapy. Should a woman become pregnant or suspect she is pregnant while participating in this study, she should inform her treating physician immediately.
   3.1.6.1 A female of child-bearing potential is any woman (regardless of sexual orientation, having undergone a tubal ligation, or remaining celibate by choice) who meets the following criteria:
   - Has not undergone a hysterectomy or bilateral oophorectomy; or
   - Has not been naturally postmenopausal for at least 12 consecutive months (i.e., has had menses at any time in the preceding 12 consecutive months).

3.1.8 Ability to understand and the willingness to sign a written informed consent
3.1.9 Eastern Cooperative Oncology Group (ECOG) performance status 0-1
3.1.10 Negative Urine β-HCG or negative serum quantitative β-HCG or within 2 weeks prior to registration for women of childbearing potential

3.2 Exclusion Criteria

3.2.1 Evidence of fixed vocal cord (Stage cT3)
3.2.2 Evidence of thyroid or soft tissue invasion (Stage cT4)
3.2.3 Evidence of positive nodal disease (Stage N1)
3.2.4 Evidence of metastatic disease (Stage M1)
3.2.5 Subjects may not be receiving any other investigational agents.
3.2.6 Non-squamous histology including lymphoma, neuroendocrine carcinoma, adenocarcinoma, or other histology.
3.2.7 Previous laryngeal surgery.
3.2.8 Previous laser therapy within one year prior to protocol treatment.
3.2.9 Previous head and neck radiation therapy involving the glottic larynx
3.2.10 Patients with collagen vascular disease, specifically dermatomyositis with a CPK level above normal or active skin rash, systemic lupus erythematosis, or scleroderma.
3.2.11 Any prior treatment with radiation therapy or chemotherapy for the currently diagnosed larynx cancer prior to registration.
3.2.12 History of another active uncontrolled malignancy at the time of study enrollment
3.2.13 Subjects must not be pregnant due to the potential for congenital abnormalities.
3.2.14 Patients smoking in excess of 2 packs of cigarettes per day.
3.2.15 ECOG performance status ≥ 2
3.2.16 Life expectancy < 3 years

4.0 TREATMENT PLAN

4.1 Treatment Dosage and Administration

4.1.1 This phase I clinical trial will begin with hemilarynx CyberKnife radiotherapy to a dose of 50 Gy delivered in 15 fractions. If this dose/fraction scheme has no dose limiting toxicity, there will be a reduction to 10 fractions to a bio-equivalent dose of 45 Gy. If this dose level is tolerated without dose limiting toxicity, there will be an additional reduction to 5 fractions to a bio-equivalent dose of 42.5 Gy. The patients will also be followed prospectively for voice quality and quality of life. The dose will be prescribed to provide at least 95% planning treatment volume (PTV) coverage.

Agent	Pre-medications; Precautions	Dose	Route	Schedule
Dexamethasone	Pre-medicate with Dexamethasone 1 hour prior to radiation treatments for dose level 1 and 2	4 mg	Oral	Daily 1 hour prior to radiation therapy

Dose-Escalation Schedule		
**Dose Level**	**Dose of the Study Agent(s)***	**Minimum Number of Patients**
Level -1	52.5 Gy in 16 fractions of 3.28 Gy per fraction daily	3
Level 0	50 Gy in 15 fractions of 3.33 Gy per fraction daily, delivered 5 fractions per week	3
Level 1	45 Gy in 10 fractions of 4.5 Gy per fraction daily, delivered 3 fractions per week	7
Level 2	42.5 Gy in 5 fractions of 8.5 Gy per fraction daily, delivered 2 fractions per week	7

Toxicity Dose Reductions	
**Event**	**Action**
**Laryngeal Edema**	
Grade 1 Asymptomatic; clinical or diagnostic observations only; intervention not indicated	None
Grade 2 Symptomatic; medical intervention indicated (e.g., dexamethasone, epinephrine, antihistamines)	None
Grade 3 Stridor; respiratory distress; hospitalization indicated	See Section 10 Statistical Considerations
Grade 4 Life-threatening airway compromise; urgent intervention indicated (e.g., tracheotomy or intubation)	Termination of trial with reporting of dose limiting toxicity
Grade 5 Death	Termination of trial with reporting of dose limiting toxicity

**Voice**	
Grade 1 Mild or intermittent change from normal voices	None
Grade 2 Moderate or persistent change from normal voice; still understandable	None
Grade 3 Severe voice changes including predominantly whispered speech; may require frequent repetition or face-to-face contact for understandability; may require assistive technology	See Section 10 Statistical Considerations
**Dyspnea**	
Grade 1 Shortness of breath with moderate exertion	None
Grade 2 Shortness of breath with minimal exertion; limiting instrumental ADL	None
Grade 3 Shortness of breath at rest; limiting self care ADL	See Section 10 Statistical Considerations
Grade 4 Life-threatening consequences; urgent intervention indicated	Termination of trial with reporting of dose limiting toxicity
Grade 5 Death	Termination of trial with reporting of dose limiting toxicity
**Stridor**	
Grade 3 Respiratory distress limiting self care ADL; medical intervention indicated	See Section 10 Statistical Considerations
Grade 4 Life-threatening airway compromise; urgent intervention indicated (e.g., tracheotomy or intubation)	Termination of trial with reporting of dose limiting toxicity
Grade 5 Death	Termination of trial with reporting of dose limiting toxicity
**Cough**	
Grade 1 Mild symptoms; nonprescription intervention indicated	None
Grade 2 Moderate symptoms, medical intervention indicated; limiting instrumental ADL	None
Grade 3 Severe symptoms; limiting self care ADL	See Section 10 Statistical Considerations
**Myelitis**	
Grade 1 Asymptomatic; mild signs (e.g., Babinski's reflex or Lhermitte's sign)	None
Grade 2 Moderate weakness or sensory loss; limiting instrumental ADL	None
Grade 3 Severe weakness or sensory loss; limiting self care ADL	See Section 10 Statistical Considerations
Grade 4 Life-threatening consequences; urgent intervention indicated	Termination of trial with reporting of dose limiting toxicity
Grade 5 Death	Termination of trial with reporting of dose limiting toxicity
**Skin and Subcutaneous Disorders**	
Grade 1 Asymptomatic or mild symptoms; clinical or diagnostic observations only; intervention not indicated	None
Grade 2 Moderate; minimal, local or noninvasive intervention indicated; limiting age-appropriate instrumental ADL	None
Grade 3 Severe or medically significant but not	See Section 10 Statistical Considerations

immediately life-threatening; hospitalization or prolongation of existing hospitalization indicated; disabling; limiting self care ADL	
Grade 4 Life-threatening consequences; urgent intervention indicated	Termination of trial with reporting of dose limiting toxicity
Grade 5 Death	Termination of trial with reporting of dose limiting toxicity
**Injury to Carotid Artery**	
Grade 3 Severe symptoms; limiting self care ADL (e.g., transient cerebral ischemia); repair or revision indicated	See Section 10 Statistical Considerations
Grade 4 Life-threatening consequences; urgent intervention indicated	Termination of trial with reporting of dose limiting toxicity
Grade 5 Death	Termination of trial with reporting of dose limiting toxicity
**Hypothyroidism**	
Grade 1 Asymptomatic; clinical or diagnostic observations only; intervention not indicated	None
Grade 2 Symptomatic; thyroid replacement indicated; limiting instrumental ADL	None
Grade 3 Severe symptoms; limiting self care ADL; hospitalization indicated	See Section 10 Statistical Considerations
Grade 4 Life-threatening consequences; urgent intervention indicated;	Termination of trial with reporting of dose limiting toxicity
Grade 5 Death	Termination of trial with reporting of dose limiting toxicity
**Hypoparathyroidism**	
Grade 1 Asymptomatic; clinical or diagnostic observations only; intervention not indicated	None
Grade 2 Moderate symptoms; medical intervention indicated	None
Grade 3 Severe symptoms; medical intervention or hospitalization indicated	See Section 10 Statistical Considerations
Grade 4 Life-threatening consequences; urgent intervention indicated	Termination of trial with reporting of dose limiting toxicity
Grade 5 Death	Termination of trial with reporting of dose limiting toxicity

Dose limiting toxicities (DLT) will be defined as occurring whenever any one of the following are manifested as occurring whenever any one of the following are manifested: laryngeal edema (grade 4 or 5), dyspnea (grade 4 or 5), stridor (grade 4 or 5) probably or definitely related to the protocol.

4.2 CT-based simulation

For CyberKnife planning, a 4-D respiratory CT-scan will be performed for target delineation and generation of GTV and internal target volume (ITV). Intravenous contrast is recommended to be administered at time of CT-simulation. During simulation, an Aquaplast mask will be custom molded for daily immobilization during radiation treatments. A head rest will be positioned beneath the patient's head and neck fitted at discretion of treating physician that will position the neck in a slightly extended position that may be reproduced daily.

4.3 Target Volumes

Carcinoma *in situ* or cT1 lesions
The gross tumor volume (GTV) and internal target volume (ITV) will be contoured based upon 4-D respiratory gated CT-scan. The clinical target volume (CTV) will include the ITV plus a 2 mm geometric expansion that may be clinically modified at the discretion of the treating physician. For lesions coming within 2 mm of or involving the ipsilateral arytenoid, the arytenoid will be included in the CTV. For lesions located within 2 mm of the anterior commissure or involving the anterior commissure, the CTV will include the anterior commissure as well as 2 mm of the adjacent contralateral vocal cord. The planning treatment volume (PTV) will be the CTV plus a 3 mm uniform expansion in all directions.

cT2 lesions
The gross tumor volume (GTV) and internal target volume (ITV) will be contoured based upon 4-D respiratory gated CT-scan. The clinical target volume (CTV) will include the ITV plus a 2 mm geometric expansion that may be modified at the discretion of the treating physician, entire ipsilateral vocal cord, and ipsilateral paraglottic space. For lesions coming within 2 mm of or involving the ipsilateral arytenoid, the arytenoid will be included in the CTV. For lesions located within 2 mm of the anterior commissure or involving the anterior commissure, the CTV will include the anterior commissure as well as 2 mm of the adjacent contralateral vocal cord. The PTV will be the CTV plus a 3 mm uniform expansion in all directions.

Bilateral cord involvement
For lesions involving bilateral vocal cords, the clinical target volume (CTV) will include the ITV with 2 mm geometric margin that may be modified at the discretion of the treating physician, bilateral vocal cords, and bilateral paraglottic spaces. The arytenoids and/or anterior commissure will only be included in the CTV if the tumor involves or comes within 2 mm of it. The PTV will be the CTV plus a 3 mm uniform expansion in all directions.

4.4 Critical Structures

The following critical structures will be contoured as OAR.

- Right carotid artery – The right carotid artery will be identified and contoured from the level of the hyoid superiorly to the level of the cricoid inferiorly
- Left carotid artery - The right carotid artery will be identified and contoured from the level of the hyoid superiorly to the level of the cricoid inferiorly
- Thyroid gland - The thyroid is easily visible on a non-contrast CT due to its preferential absorption of Iodine, rendering it “brighter” or denser than the surrounding neck soft tissues. The left and right lobes of the thyroid are somewhat triangular in shape, and often do not converge anteriorly at mid-line. All “bright” thyroid tissue should be contoured.
- Anterior commissure – The anterior commissure will be defined as the 1 mm laterally along the true vocal cords from the midline of the anterior cords, and will be contoured unless gross tumor involves it or comes within 2 mm of it
- Ipsilateral arytenoid – The ipsilateral arytenoid of the laryngeal skeleton will be identified and contoured unless gross tumor involves or comes within 2 mm of it
- Contralateral Arytenoid – The contralateral arytenoid of the laryngeal skeleton will be identified and contoured
- Contralateral vocal cord – Unless there is bilateral vocal cord involvement, the contralateral true vocal cord will be identified and contoured

- Larynx – The entire laryngeal skeleton will be contoured including the thyroid cartilage, epiglottis, cricoids, and cuneiform cartilages.
- Spinal cord – The spinal cord will be contoured from 2 cm superior to 2 cm inferior from the larynx
- Skin – The skin will be contoured based upon an external contour of the body

4.5 Dose Constraints

OAR	Volume	Dose
Thyroid	Mean	<10% prescribed dose
Right carotid artery	Maximum point	<10% prescribed dose
Left carotid artery	Maximum point	<10% prescribed dose
Spinal cord	Maximum point	<5% prescribed dose
Skin	Maximum point	<10% prescribed dose

4.6 Radiation Adverse Events

The consequences of laryngeal toxicity and cough will be monitored and graded according to the Common Terminology Criteria for Adverse Effects (CTCAE) v4.

4.7 Serious Adverse Event Reporting

Adverse Events: Definitions and Reporting

Adverse Events will be reported as indicated by the appropriate following table (see below).

Definition

An adverse event is defined as any untoward or unfavorable medical occurrence in a human research study participant, including any abnormal sign (for example, abnormal physical exam or laboratory finding), symptom, clinical event, or disease, temporarily associated with the subject's participation in the research, whether or not it is considered related to the subject's participation in the research.

Adverse events encompass clinical, physical and psychological harms. Adverse events occur most commonly in the context of biomedical research, although on occasion, they can occur in the context of social and behavioral research. Adverse events may be expected or unexpected.

Severity

Adverse events will be graded by a numerical score according to the defined NCI Common Terminology Criteria for Adverse Events (NCI CTCAE) and version number specified in the protocol. Adverse events not specifically defined in the NCI CTCAE will be scored on the Adverse Event log according to the general guidelines provided by the NCI CTCAE and as outlined below.

- Grade 1: Mild
- Grade 2: Moderate
- Grade 3: Severe or medically significant but not immediately life threatening

- Grade 4: Life threatening consequences
- Grade 5: Death related to the adverse event

Serious Adverse Events

ICH Guideline E2A and the UTSW IRB define serious adverse events as those events, occurring at any dose, which meets any of the following criteria:

- Results in death
- Immediately life-threatening
- Results in inpatient hospitalization or prolongation of existing hospitalization
- Results in persistent or significant disability/incapacity
- Results in a congenital anomaly/birth defect
- Based upon appropriate medical judgment, may jeopardize the subject's health and may require medical or surgical intervention to prevent one of the other outcomes listed in this definition.

Note: A "Serious adverse event" is by definition an event that meets any of the above criteria. Serious adverse events may or may not be related to the research project. A serious adverse event determination does not require the event to be related to the research. That is, both events completely unrelated to the condition under study and events that are expected in the context of the condition under study may be serious adverse events, independent of relatedness to the study itself. As examples, a car accident requiring overnight hospitalization would be a serious adverse event for any research participant; likewise, in a study investigating end-stage cancer care, any hospitalization or death would be a serious adverse event, even if the event observed is a primary clinical endpoint of the study. Refer to the UTSW IRB website at http://www.utsouthwestern.net/intranet/research/research-administration/irb/study-management/adverse-events.html to determine when a serious adverse event requires reporting to the IRB.

Unanticipated Problems:

The term "unanticipated problem" is found, but not defined in the regulations for the Protection of Human Subjects at 45 CFR 46, and the FDA regulations at 21 CFR 56. Guidance from the regulatory agencies considers unanticipated problems to include any incident, experience, or outcome that meets each of the following criteria:

- Unexpected (in terms of nature, severity or frequency) AND
- Definitely, probably, or possibly related to participation in the research AND
- Serious or a possible unexpected problem in that the research places subjects or others at greater risk of harm than was previously known or recognized. Note: Any serious adverse event would always suggest a greater risk of harm.

Follow-up

All adverse events will be followed up according to good medical practices.

Reporting

Local unanticipated problems require expedited reporting, and are submitted to the UTSW IRB through the UTSW eIRB and to the SCC DSMC Coordinator. Hardcopies or electronic versions of the eIRB report; FDA Form #3500A forms, or other sponsor forms, if applicable; and/or any other supporting documentation available should be forwarded to the DSMC Coordinator. The DSMC Coordinator forwards the information onto the DSMC Chairman who determines if immediate action is required. Follow-up eIRB reports, and all subsequent SAE documentation

that is available are also submitted to the DSMC Chair who determines if further action is required.

All local serious adverse events which occur on research subjects on protocols for which the SCC is the DSMC of record require reporting to the DSMC regardless of whether IRB reporting is required. Hardcopies or electronic versions of the FDA Form #3500A forms, or other sponsor forms, if applicable; and/or any other supporting documentation available should be forwarded to the DSMC Coordinator.

If the event occurs on a multi-institutional clinical trial coordinated by the Cancer Center, the DOT Manager or lead coordinator ensures that all participating sites are notified of the event and resulting action, according to FDA guidance for expedited reporting. DSMC Chairperson reviews all serious adverse events within upon receipt from the DSMC Coordinator. The DSMC Chairperson determines whether action is required and either takes action immediately, convenes a special DSMC session (physical or electronic), or defers the action until a regularly scheduled DSMC meeting.

Participating sites of multi-institutional clinical trials coordinated by the UTSW Radiation Oncology:

Written reports to

UTSW Radiation Oncology Study Coordinator or Clinical Research Manager within 1 working day
Email: jean.wu@utsouthwestern.edu or susan.cooley@utsouthwestern.edu

Fax: 214-645-8913

UTSW Radiation Oncology Study Coordinator or Clinical Research Manager will report to UTSW SCC Data Safety Monitoring Committee Coordinator

UTSW SCC Data Safety Monitoring Committee Coordinator
Email: SCCDSMC@utsouthwestern.edu
Fax: 214-648-7018 or deliver to NB 2.418

UTSW Institutional Review Board (IRB)

Submit via eIRB with a copy of the final sponsor report as attached supporting documentation

1. SAEs
   Local serious adverse events (SAEs) for studies where SCC DSMC is the DSMC of record require reporting to the DSMC coordinator within 2 working days of PI awareness, or as described in the protocol.

2. Unanticipated Problems
   - Local unanticipated problems require reporting to the UTSW IRB within 2 working days of PI awareness of the event.
   - Unanticipated problems, including those that occur as non-local events, require reporting to the UTSW IRB within 10 working days of PI awareness of the event.

For further guidance for Investigators regarding safety reporting requirements for INDs and BA/BE studies, refer to FDA Draft Guidance document:
http://www.fda.gov/downloads/Drugs/GuidanceComplianceRegulatoryInformation/Guidances/UCM227 351.pdf

Attribution of an event can be categorized as: unrelated, unlikely, possible, probable, or definitely related.

Adverse events (below grade 3) do not need to be submitted immediately. Rather, they should be documented in the Adverse Events CRF along with a brief description of the event, grade, and attribution).

4.8 Concomitant Medications/Treatments

Concomitant use of immunosuppressant agents such as inhibitors of the mammalian target of rapamycin (mTOR), chronic high-dose steroids, small-molecule immune modulating agents will be prohibited. Anti-coagulant medications such as Coumadin, Heparin, Lovanox, Agatroban or other agents affecting the coagulation cascade will be prohibited.

4.9 Other Procedures

For dose level 1 and 2, a dexamethasone 4 mg oral tablet will be administered one hour prior to initiation of radiation therapy.

4.10 Duration of Therapy

The end of protocol therapy will occur if:

- Inter-current illness that prevents further administration of treatment
- Unacceptable adverse event(s)
- Subject decides to withdraw from the study, or
- General or specific changes in the patient's condition render the subject ineligible for further treatment at the discretion of the investigator or treating physician

4.11 Duration of Follow-Up

Subjects will be followed for 5 years after completion of radiation therapy or until death, whichever occurs first. Subjects removed from treatment for unacceptable adverse events will be followed until resolution or stabilization of the adverse event. First follow-up after completion of treatment will occur at four weeks. Thereafter, protocol required follow-up in the Radiation Oncology Clinic or Otolaryngology Clinic will occur at every 3 months for the first year, every 6 months for the second year, and annually for years 3, 4, and 5 following treatment.

4.12 Removal of Subjects from Protocol Therapy

Subjects will be removed from therapy if dose limiting toxicity is experienced. The Principal Investigator will be notified, and reason documented for study removal and the date the subject was removed in the Case Report Form. The subject should be followed-up per protocol.

4.13 Subject Replacement

Three subjects within dose levels -1 and 0 must be observed for 90 days before accrual to the next higher dose level may begin. For level 1, 7 patients must be observed for 90 days before accrual to the next higher dose level. If a subject is withdrawn from the study prior to completing therapy without experiencing a dose limiting toxicity prior to withdrawal, an additional subject may be added to that dose level. Subjects missing 3 or more radiation treatments due to toxicity will not be replaced since these subjects will be considered to have experienced a dose limiting toxicity.

4.14 R.T. Quality Assurance Reviews

Dr. Timmerman, along with a medical physicist, will perform an RT Quality Assurance Review after complete data for the first 15 cases enrolled has been obtained. They will perform the next review after complete data for the next 15 cases enrolled has been obtained. The final cases will be reviewed within 3 months after this study has reached the target accrual or as soon as complete data for all cases enrolled has been received, whichever occurs first.

5.0 STUDY PROCEDURES

5.1 Screening/Baseline Procedures

Assessments performed exclusively to determine eligibility for this study will be done only after obtaining informed consent. Assessments performed for clinical indications (not exclusively to determine study eligibility) may be used for baseline values even if the studies were done before informed consent was obtained.

All screening procedures must be performed within 30 days prior to registration unless otherwise stated. The screening procedures include:

5.1.1 Informed Consent

5.1.2 Medical history

Complete medical and surgical history, history of infections

5.1.3 Demographics

Age, gender, race, ethnicity

5.1.4 Review subject eligibility criteria

5.1.5 Review previous and concomitant medications

5.1.6 Physical exam including vital signs, height and weight

Vital signs (temperature, pulse, respirations, blood pressure), height, weight

5.1.7 Performance status

Performance status evaluated prior to study entry according to the ECOG

5.1.8 Adverse event assessment

All acute and late adverse events from protocol radiation therapy will be reported and scored for severity using the NCI Common Terminology Criteria for Adverse Events (CTCAE) version 4.0. A copy of the CTCAE v4.0 can be downloaded from the CTEP home page (http://ctep.info.nih.gov).

5.1.1 Laboratory work - Pregnancy test (for females of child bearing potential)

All females of childbearing potential will undergo either a urine qualitative β-HCG level or serum quantitative β-HCG level to show that they are not currently pregnant.

5.1.2 Tumor assessment

Tumor assessment will be performed by physical examination, CT-scan of the neck, and flexible laryngoscopy.

5.1.3 QoL Questionnaires and Symptom Questionnaires

EORTC QLQ-C30, H&N 35, MDADI, VHI, EQ-5D, and cost and convenience questionnaire. These forms will be referred to collectively as QoL Questionnaires described in section 6**.**

5.2 Procedures during Treatment

5.2.1 Prior to Each Treatment Cycle

- Clinical Assessment, vital signs

5.2.2 Day 1

- Initiation of CyberKnife radiotherapy
- Clinical Assessment

5.2.3 Weekly during treatment (as applicable for fractionation)

- Clinical Assessment
- Toxicity Assessment

5.2.4 Four weeks after treatment termination

- Interim history
- Physical exam, vital signs
- Flexible laryngoscopy to assess tumor response and toxicity
- Quality of life and voice quality assessment

5.3 Follow-up Procedures

After initial follow-up at four weeks after completion of radiation therapy, subjects will be followed every 3 months for the first year, every 6 months for the second year, and annually for years 3, 4, and 5. These protocol mandated procedures will occur at each follow-up:

- Interim history
- Physical exam, vital signs
- Laryngoscopy to assess tumor response and toxicity
- Quality of life and voice quality assessment
- CT-scan of neck with tumor measurements

5.4 Time and Events Table

	Pre-study	Day 1	Weekly	4 weeks after completion	Follow-up *B
Assessment		X	X	X	
Informed Consent	X				
History and Physical Exam	X			X	X
Performance Status	X	X		X	X
Toxicity (include DLT) Evaluations		X	X	X	X
CT-scan based tumor measurements	X				X
Laryngoscopy	X			X	X
Chest x-ray or Chest CT-scan	X				
Urine β-HCG (females)	X				
Thyroid stimulating hormone (TSH)	X				X (yearly)
Patient Cost and Convenience Form	X				
HR-QoL/Utility Forms *A<br>1) EQ-5D<br>2) EORTC-QLQ C30 + HN 35<br>3) VHI<br>4) MDADI<br>5) Cost and convenience questionnaire (*A)	X				X
				X	X
	X			X	X

* A. Cost and Convenience Questionnaire will only be administered at the first available follow up. All other QoL surveys will take place as stated per this row.

*B. After initial follow-up at 4 weeks after completion of radiation therapy, subjects will be followed every 3 months for the first year, every 6 months for the second year, and annually for years 3, 4, and 5. These protocol mandated procedures will occur at each follow-up:

- Interim history
- Physical exam, vital signs
- Laryngoscopy to assess tumor response and toxicity
- Quality of life and voice quality assessment
- CT-scan of neck with tumor measurements

5.5 Removal of Subjects from Study

Subjects can be taken off the study treatment and/or study at any time at their own request, or they may be withdrawn at the discretion of the investigator for safety, behavioral or administrative reasons. The reason(s) for discontinuation will be documented and may include:

5.5.1 Subject voluntarily withdraws from treatment (follow-up permitted);

5.5.2 Subject withdraws consent (termination of treatment and follow-up);

5.5.3 Subject is unable to comply with protocol requirements;

5.5.4 Subject demonstrates disease progression (unless continued treatment with study drug is deemed appropriate at the discretion of the investigator);

5.5.5 Subject experiences toxicity that makes continuation in the protocol unsafe

5.5.6 Treating physician judges continuation on the study would not be in the subject's best interest;

5.5.7 Subject becomes pregnant (pregnancy to be reported along same timelines as a serious adverse event);

5.5.8 Development of second malignancy (except for basal cell carcinoma or squamous cell carcinoma of the skin) that requires treatment, which would interfere with this study

5.5.9 If a research subject cannot be located to document survival after a period of 2 years, the subject may be considered "lost to follow-up." All attempts to contact the subject during the two years must be documented and approved by the Data Monitoring Committee.

6.0 Data and Safety Monitoring Plan

External Data and Safety Monitoring Board

The UTSW Simmons Cancer Center (SCC) Data Safety Monitoring Committee (DSMC) is the external review of trial-related documentation, which may include detailed assessment of subject records, regulatory and pharmacy review or may be more focused depending on the nature and reason for the audit. External reflects an independent reviewer within the CRO who is external to the DOT. DSMC audits are performed by the Quality Assurance and Education Coordinator (QAC) with the overall purpose of reviewing the conduct of a clinical trial to ensure quality results. The audit schedule prioritizes investigator-initiated institutional therapeutic trials and frequency is determined by the risk of the trial. Audits of other trial types are performed according to the risk level assigned to the study. The schedule is designed to ensure that every disease site will have trials audited at least annually.

The SCC DSMC is responsible for monitoring data quality and patient safety for all UTSW SCC clinical trials. As part of that responsibility, the DSMC reviews all local serious adverse events and unanticipated problems in real time as they are reported and reviews adverse events on a quarterly basis. The quality assurance activity for the Clinical Research Office provides for periodic auditing of clinical research documents to ensure data integrity and regulatory compliance.

A detailed description of the Data and Safety Monitoring Plan is available the Clinical Research Office Operations Manual.

The SCC DSMC meets quarterly and conducts annual comprehensive reviews of ongoing clinical trials, for which it serves as the DSMC of record. The Quality Assurance and Education Coordinator (QAC) works as part of the DSMC to conduct regular audits based on the level of risk. Audit findings are reviewed at the next available DSMC meeting. In this way, frequency of DSMC monitoring is dependent upon the level of risk. Risk level is determined by the DSMC Chairman and a number of factors such as the patient population to be studied; adequacy of the data management system; and procedures to ensure the safety of study subjects based on the associated risks of the study. Protocol-specific DSMC plans must be consistent with these

principles. The following examples can be used to begin developing DSMC plans for individual protocols.

- High risk examples include Phase I, II or III studies where the SCC is the DSMC; gene therapy or recombinant DNA studies; and studies in which the investigator holds the IND.
- Moderate risk examples include pilot studies and other Phase I, II or III studies where the SCC DSMC is not the DSMC of record and which involve a non-FDA approved drug.
- Low risk examples include Phase II or III studies involving FDA-approved drugs where the SCC DSMC is not the DSMC of record, or trials, which involve non-therapeutic interventions.
- Exempt examples include non-interventional studies, which are exempt from audit requirements outlined above.

Internal Data and Safety Monitoring Committee

The Radiation Oncology Clinical Research Office (CRO) reports serious adverse events (SAEs) to Radiation Oncology Data Safety Monitoring Committee (DSMC) monthly. These SAEs are also reported to the University of Texas Southwestern Medical Center (UTSW) IRB per IRB guidelines and SCC DSMC.

All clinical trials are reviewed on monthly basis for enrollment. These trials are assessed for safety on a continual basis throughout the life of the trial. For investigator-initiated trials, all SAEs are monitored – both local and at affiliated institutions.

7.0 Data Collection

Data should be submitted to:

Department of Radiation Oncology
Clinical Research Office
The University of Texas Southwestern Medical Center
Attention: Jean Wu, Project Manager
5801 Forest Park Road
Dallas, Texas 75390-9183
FAX #: 214-645-8913

Patients will be identified only by initials (first middle last) and a unique study ID number assigned to each study participant; if there is no middle initial, a hyphen will be used (first-last). Last names with apostrophes will be identified by the first letter of the last name.

8.0 Quality of Life

8.1.1 EORTC QLQ C30 and H&N 35

"European Organization for Research and Treatment of Cancer 30 (EORTC QLQ C-30)" is a 30 (10 minutes) question quality of life questionnaire that has been used in several current and prior European studies to assess quality of life in patients undergoing treatment for cancer. We will only collect this questionnaire in patients treated to lung, liver, and pancreatic sites based on the extensive European quality of life data which will serve as a general comparison for these disease sites. EORTC QLQ questionnaires are standard of care for RTOG trials in the Europe. The H&N 35 (10 minutes) is an additional add-on questionnaire exploring side effects commonly experienced by patients treated with radiation therapy and/or chemotherapy for head and neck cancers.

8.1.2 EQ-5D

The "EQ-5D" is a patient self-administrated questionnaire that takes approximately 5 minutes to complete (See appendix for form). The first part consists of 5 items covering 5 dimensions including: mobility, self care, usual activities, pain/discomfort, and anxiety/depression. Each dimension can be graded on 3 levels: 1-no problems, 2-moderate problems, and 3-extreme problems. Health states are defined by the combination of the leveled responses to the 5 dimensions, generating 243 health states to which unconsciousness and death are added.

The 5-item index score is transformed into a utility score between 0, "Worst health state," and 1, "Best health state." The index score or the cost-utility equation can be used in the quality adjusted survival analysis depending on the health state(s) of interest.

8.2 Cost effectiveness data collection:

Health care utilization data needed to assess costs will be obtained from treatment records. Additionally, in order to assess the treatment related indirect costs and patient out of pocket costs, a form will be administered during the last week of treatment or at the first available follow up visit after completion of radiation treatment (see Appendix VII).

- Hospitalizations: For hospitalizations with physician billing records, impatient physician costs will be estimated by applying Medicare payment rates under the RBRVS-based Medicare Fee Schedule to billed procedures in the physician billing records.
- Treatment Cost: Direct costs of radiation treatment including consultation, simulation, treatment planning, and treatment delivery. Patient bills related to treatment will be obtained and estimated by total billed charges adjusted by facility-specific cost-to-charge ration from Medicare cost reports as described above.
- Emergency Room visits: The date of ER visit and name of the facility, and whether the ER visit resulted in a hospital admission. ER costs will be estimated using Medicare average payment rates for facility and physician charges, using the merged MEDPAR and MBS data as described above.
- Physician and Clinic Visits: The date of the visit, the name of the physician or physician clinic, and the service provided (physician exam, lab test, physical therapy, etc.). Costs for physician and clinic visits will be calculated based on billing records obtained for such visits, using Medicare payment rates for procedures indicated in the clinic billing records.
- Medications: Prescription drugs used, including dosage strength and frequency of administration. Information about name, dose, and frequency of all prescription medications will be recorded. The medications used by the study patients will be assigned an NDC drug code. Unit costs for these drugs will be estimated as the "AWP" price published in the Red Book less 15%. Outpatient drug costs will be calculated by multiplying unit cost by the number of pills used per day times the length of time the patient received the medication. Note that costs of drugs administered through a clinic (e.g., reimbursed under Medicare Part B) are included under "clinic visit costs" and impatient drug costs are included under "inpatient facility costs."

9.0 QUALITY OF LIFE AND COST-EFFECTIVENESS

9.1 Health-Related Quality of Life (HRQOL) Analysis

The study design is to prospectively analyze the HRQOL among patients with early stage glottis cancer who are treated on this protocol. While hypofractionation is hypothesized to yield greater tumor cell kill, it may also increase the normal tissue toxicity, in which case there may be a decrease in HR-QOL. The primary normal tissue toxicities in patients receiving radiation

depends on the location of the treatment. Prior studies have demonstrated that the most sensitive and clinically meaningful method for accurately capturing the normal tissue toxicities is via patients reported outcomes (PROs), such as HRQOL.

In this non-randomized trial, we plan to assess the HR-QoL forms at specific time points to minimize patient burden: baseline (pretreatment), at the end of radiation, and at subsequent follow ups (See Appendix).

In addition, the EQ-5D will be used as well. EQ-5D is a standardized instrument for use as a measure of health outcome. Applicable to a wide range of health conditions and treatments, it provides a simple descriptive profile and a single index value for health status. The US version of the EQ-5D will be used, to enable mapping of general HR-QoL scores from EQ-5D scores into health state utility scores (ranging from 0 to 1) for the US population. These utility scores are needed for cost-utility analysis (estimates of costs per "quality adjusted" life-year gained) [42,43].

HRQoL of patients with early stage glottis cancer is fortunately well documented but there exist wide variability in the forms used to assess HR-QoL and the majority of HR-QoL being reported for traditional fractionated radiotherapy, surgery, or laser techniques.

In a study from the Czech Republic evaluating the health-related quality of life of patients treated with either radiotherapy or endoscopic trans oral indo laryngeal surgery, the authors concluded that was no difference between patients treated with the cordectomy and radiotherapy. They assessed health related quality of life using the EORTC-QLQ C30 and H&N 35 forms [39].

With the improved utilization of endoscopic laser surgery, a similar study assess the quality of life of patients undergoing laser surgery versus radiotherapy. There health-related quality of life was assessed via EORTC-QLQ C30 and H&N 35 forms. The results of this study showed that both modalities achieved excellent quality of life in the treatment of early laryngeal tumors [44].

While numerous other studies use a wide range of questionnaires to assess health-related quality of life, we chose to use the EORTC-QLQ C30 and H&N 35 similar to the above-mentioned trials to aid in future comparisons.

All HR-QoL will be used for descriptive purposes only due the single arm phase I nature of this protocol.

Additionally, in order to calculate the indirect costs associated with hypofractionated radiation treatment, a single administration of a short economic questionnaire will take place at the end of radiation treatment or first available follow up or whichever occurs first. This questionnaire which has been adapted for administration in the United States has been used in economic assessments in rural Canadian cancer health service research [45].

9.2 Cost-Effectiveness Analysis (CEA)

For the primary CEA analysis, we will estimate cost accumulated within 1 years.

Since patients are enrolled into the study over time and some patients are still alive at the end of the study, their survival time and costs are censored. Due to the presence censoring, we cannot use a simple average of the patients' total costs, a simple average of the patients' costs for those with complete cost information, or a Kaplan-Meier estimator on censored costs, since these all produce biased estimators of the mean costs[42]. Instead, we will use the inverse-probability weighting method to calculate average costs.[43,46] The assumption used in this method is that censoring is independent of the survival time, or cost collection

process, which is often satisfied in well-conducted clinical trials. If the new treatment can both extend patients' survival time (or quality-adjusted survival time), and save costs at the same time, the new treatment will be preferred to the current standard treatment under any willingness to pay threshold.

However, if the new treatment extends survival time but costs more, cost-effectiveness analysis provides an estimate of the incremental cost of greater incremental effectiveness. For traditional cost-effectiveness analysis, treatment effectiveness is measured simply as survival time. The incremental cost-effectiveness ratio indicates the additional cost required to attain one additional year of survival. For cost-utility analysis, treatment effectiveness is measured as quality-adjusted survival time (which accounts for the impact of treatment on both mortality and morbidity, including any differences in adverse affects of treatment affecting HR-QoL). For cost-utility analysis, the incremental cost-effectiveness ratio indicates the additional cost required to attain one additional year of quality adjusted survival.

9.3 Projection Model and Sensitivity Analysis

If the new treatment is implemented in usual practice, some of its potential benefits to patients may extend beyond the time horizon of the clinical trial. We will explore the potential to use results from the clinical trial based cost-effectiveness analysis, augmented with information from secondary sources, to develop a model to project costs and effectiveness beyond the time horizon included in the clinical trial. Any such model projections would be subjected to probabilistic sensitivity analysis, to assess the impact of parameter uncertainty on estimated cost effectiveness results. This is typically done via Markov Modeling with probabilistic sensitivity analysis.

Additionally, this Markov model will allow us to perform a value of information (VOI) analysis in order to assess from cost-effectiveness standpoint which variable(s) (i.e. voice quality, recurrence, salvage, HR-QoL) are most important to assess the future clinical trials.

9.4 Quality adjusted survival time:

The quality-adjusted survival time estimates need to account for the presence of censoring. Due to the induced informative censoring problem, the ordinary survival method (e.g., Kaplan-Meier estimator) cannot be applied in this case [43,46,47]. Accordingly, we will use the inverse-probability weighted method of Zhao and Tsiatis to carry out the survival time analysis [43,46]. To estimate quality adjusted survival time, data from EQ-5D will first be translated into utility measures. These measures are obtained at discrete time points, so they will be interpolated into the time intervals between the visits. The quality-adjusted survival time is just an integration of the utility measures over a patient's survival time, or until the time limit similar as the cost calculation, whichever occurs earlier.

10.0 Statistical Considerations

10.1 Primary endpoint

The primary endpoint of this phase I clinical trial will be to either reach the minimum number of fractions that is tolerated for larynx irradiation or a dose of 42.5 Gy in 5 fractions whichever comes first. Patients will be treated in cohorts of 3-15. Toxicity will be graded using the National Cancer Institute (NCI) Common Toxicity Criteria for Adverse Events (CTCAE) version 4.0. A dose-limiting toxicity (DLT) is a grade 3 or greater toxicity definitely related to treatment in the following categories: laryngeal edema, voice, dyspnea, striodor, or cough. All reported DLTs will be verified by the study

chair, data monitoring committee, and as appropriate, independent review before final determination that a DLT has in fact occurred. The number of fractions will be reduced from 15, to 10 fractions, and then to 5 fractions (reduced by 5 fractions per step). The phase 1 portion of the study will be completed with either of the following events occur: 1) the MTD for a cohort is reached or 2) when the lowest number of fractions is treated and tolerated (42.5 Gy in 5 fractions).

10.2 Phase 1 waiting periods

Fraction reduction for this phase 1 clinical trial will not occur until sufficient waiting has occurred after a cohort has been treated. A period of 90 days must pass before further fraction reduction for the purpose of assessing toxicity. If 90 days have passed without a DLT at a dose level, the trial will be allowed to the next level, so long as either criteria for defining the MTD or criteria for further fraction reduction is not reached. If the maximum number of patients are enrolled to a given fraction level, yet criteria for follow-up is not reached in a representative sample of patients, further enrollment to the protocol will be suspended until adequate follow-up is reached. No action will be taken for Grade 1 or Grade 2 toxicities. If any 2 Grade 3 toxicities occur within a dose level likely or definitely related to radiation therapy, the trial will be terminated with reporting of DLT at that dose level. Any Grade 4 or Grade 5 toxicity found to be likely or definitely related to treatment will result in termination of the trial with reporting of DLT at that dose level.

Grade 3 toxicities likely or definitely related to protocol therapy will result in action in schema provided below:

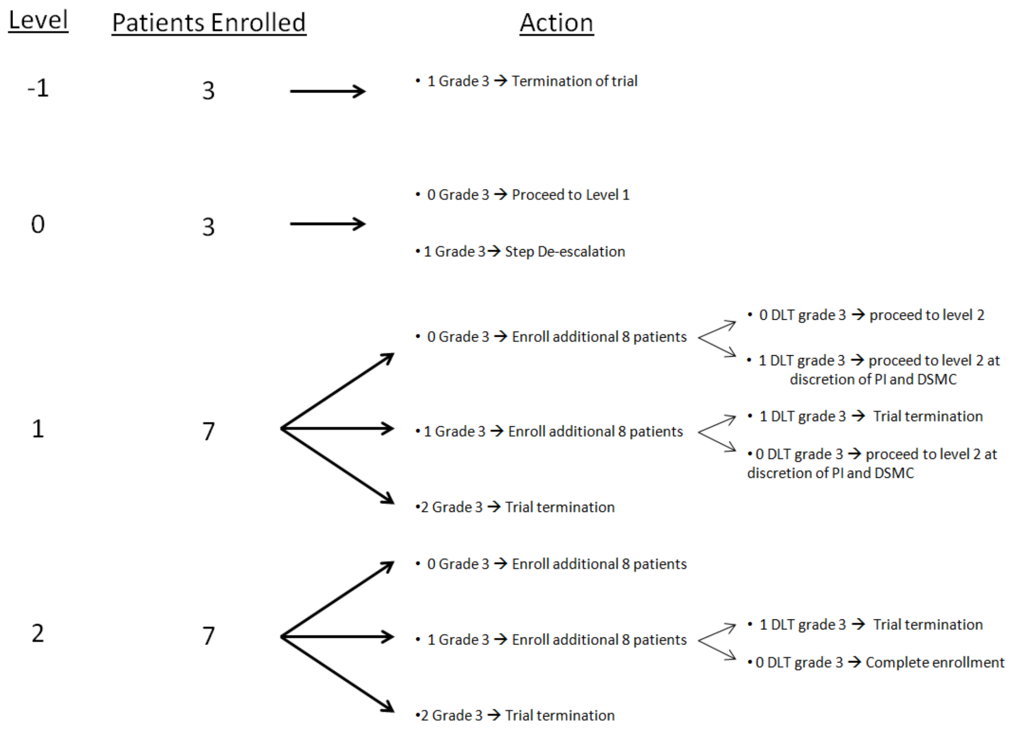

For level -1, if a Grade 3 toxicity occurs, the trial will be terminated with reporting of DLT.

For level 0, if a Grade 3 toxicity occurs, a step de-escalation will occur to level -1. If no grade 3 toxicities are experienced at level 0, the trial will proceed to level 1.

For level 1, if there are no Grade 3 toxicities after initial enrollment of 7 patients, an additional 8 patients will be enrolled. If there is no grade 3 toxicity after enrollment of an additional 8 patients, the trial will proceed to level 2. If there is a 1 grade 3 toxicity in the additional 8 patients, the trial will proceed to level 2 at the discretion of the PI and DSMC.

For level 1, if there is 1 Grade 3 in the initial 7 patient cohort, an additional 8 patients will be enrolled. If there is an additional Grade 3 toxicity at this level, the trial will be terminated with reporting of DLT. If there are no further grade 3 toxicities in the additional 8 patients, proceeding to the next level will be done at discretion of the DSMC and PI.

For level 1, if there are 2 grade 3 toxicities in the initial 7 patient cohort, the trial will be terminated with reporting of DLT.

For level 2, if there are 0 grade 3 toxicities in the initial 7 patient cohort, the trial will enroll an additional 8 patients.

For level 2, if there is 1 grade 3 toxicity in the initial 7 patient cohort, the trial will enroll an additional 8 patients. If there is another grade 3 toxicity, the trial will be terminated with reporting of DLT.

For level 2, if there are 2 grade 3 toxicities in the initial 7 patient cohort, the trial will be terminated with reporting of DLT.

10.3 Secondary endpoints

Secondary endpoints for the phase 1 portion of this study include local control, overall survival, functional voice quality, cost-effectiveness, quality of life, and late toxicity (defined as toxicities occurring > 18 months post-treatment).

References

1. Siegel R, Naishadham D, Jemal A. Cancer statistics, 2013. *CA Cancer J Clin.* Jan 2013;63(1):11-30.
2. Mendenhall WM, Parsons JT, Stringer SP, Cassisi NJ, Million RR. The role of radiation therapy in laryngeal cancer. *CA Cancer J Clin.* May-Jun 1990;40(3):150-165.
3. Parsons JT, Mendenhall WM, Stringer SP, Cassisi NJ, Million RR. Radiotherapy Alone for Moderately Advanced Laryngeal Cancer (T2-T3). *Semin Radiat Oncol.* Jul 1992;2(3):158-162.
4. Rubinstein M, Armstrong WB. Transoral laser microsurgery for laryngeal cancer: a primer and review of laser dosimetry. *Lasers Med Sci.* Jan 2011;26(1):113-124.
5. Ganzer U. [Prognosis of laryngeal cancer since 1960. Results of a literature review and a survey]. *Laryngorhinootologie.* Jan 1990;69(1):1-5.
6. Pfister DG, Ang K, Brockstein B, et al. NCCN Practice Guidelines for Head and Neck Cancers. *Oncology (Williston Park).* Nov 2000;14(11A):163-194.
7. Mendenhall WM, Amdur RJ, Morris CG, Hinerman RW. T1-T2N0 squamous cell carcinoma of the glottic larynx treated with radiation therapy. *J Clin Oncol.* Oct 15 2001;19(20):4029-4036.
8. Wetmore SJ, Key JM, Suen JY. Laser therapy for T1 glottic carcinoma of the larynx. *Arch Otolaryngol Head Neck Surg.* Aug 1986;112(8):853-855.
9. Ogura JH. Management of early cancer of the vocal cord--laryngofissure, hemilaryngectomy, irradiation. *Trans Pac Coast Otoophthalmol Soc Annu Meet.* 1963;44:245-247.
10. Gowda RV, Henk JM, Mais KL, Sykes AJ, Swindell R, Slevin NJ. Three weeks radiotherapy for T1 glottic cancer: the Christie and Royal Marsden Hospital Experience. *Radiother Oncol.* Aug 2003;68(2):105-111.
11. Wiernik G, Alcock CJ, Bates TD, et al. Final report on the second British Institute of Radiology fractionation study: short versus long overall treatment times for radiotherapy of carcinoma of the laryngo-pharynx. *Br J Radiol.* Mar 1991;64(759):232-241.
12. Paterson R. Studies in optimum dosage. *Br J Radiol.* Oct 1952;25(298):505-516.
13. Rudoltz MS, Benammar A, Mohiuddin M. Prognostic factors for local control and survival in T1 squamous cell carcinoma of the glottis. *Int J Radiat Oncol Biol Phys.* Aug 1 1993;26(5):767-772.
14. Yamazaki H, Nishiyama K, Tanaka E, Koizumi M, Chatani M. Radiotherapy for early glottic carcinoma (T1N0M0): results of prospective randomized study of radiation fraction size and overall treatment time. *Int J Radiat Oncol Biol Phys.* Jan 1 2006;64(1):77-82.
15. Osman SO, de Boer HC, Heijmen BJ, Levendag PC. Four-dimensional CT analysis of vocal cords mobility for highly focused single vocal cord irradiation. *Radiother Oncol.* Oct 2008;89(1):19-27.
16. Osman SO, Astreinidou E, de Boer HC, et al. IMRT for image-guided single vocal cord irradiation. *Int J Radiat Oncol Biol Phys.* Feb 1 2012;82(2):989-997.
17. Levendag PC, Teguh DN, Keskin-Cambay F, et al. Single vocal cord irradiation: a competitive treatment strategy in early glottic cancer. *Radiother Oncol.* Dec 2011;101(3):415-419.
18. Blomgren H, Lax I, Naslund I, Svanstrom R. Stereotactic high dose fraction radiation therapy of extracranial tumors using an accelerator. Clinical experience of the first thirty-one patients. *Acta Oncol.* 1995;34(6):861-870.
19. Uematsu M, Shioda A, Suda A, et al. Computed tomography-guided frameless stereotactic radiotherapy for stage I non-small cell lung cancer: a 5-year experience. *Int J Radiat Oncol Biol Phys.* Nov 1 2001;51(3):666-670.
20. Lax I, Blomgren H, Naslund I, Svanstrom R. Stereotactic radiotherapy of malignancies in the abdomen. Methodological aspects. *Acta Oncol.* 1994;33(6):677-683.
21. Timmerman R, Papiez L, McGarry R, et al. Extracranial stereotactic radioablation: results of a phase I study in medically inoperable stage I non-small cell lung cancer. *Chest.* Nov 2003;124(5):1946-1955.
22. Wulf J, Haedinger U, Oppitz U, Thiele W, Mueller G, Flentje M. Stereotactic radiotherapy for primary lung cancer and pulmonary metastases: a noninvasive treatment approach in medically inoperable patients. *Int J Radiat Oncol Biol Phys.* Sep 1 2004;60(1):186-196.
23. Timmerman R, Paulus R, Galvin J, et al. Stereotactic body radiation therapy for inoperable early stage lung cancer. *JAMA.* Mar 17 2010;303(11):1070-1076.

24. Potters L, Steinberg M, Rose C, et al. American Society for Therapeutic Radiology and Oncology and American College of Radiology practice guideline for the performance of stereotactic body radiation therapy. *Int J Radiat Oncol Biol Phys.* Nov 15 2004;60(4):1026-1032.

25. Wolbarst AB, Chin LM, Svensson GK. Optimization of radiation therapy: integral-response of a model biological system. *Int J Radiat Oncol Biol Phys.* Oct 1982;8(10):1761-1769.

26. Yaes RJ, Kalend A. Local stem cell depletion model for radiation myelitis. *Int J Radiat Oncol Biol Phys.* Jun 1988;14(6):1247-1259.

27. Coon D, Gokhale AS, Burton SA, Heron DE, Ozhasoglu C, Christie N. Fractionated stereotactic body radiation therapy in the treatment of primary, recurrent, and metastatic lung tumors: the role of positron emission tomography/computed tomography-based treatment planning. *Clin Lung Cancer.* Jul 2008;9(4):217-221.

28. King CR, Brooks JD, Gill H, Pawlicki T, Cotrutz C, Presti JC, Jr. Stereotactic body radiotherapy for localized prostate cancer: interim results of a prospective phase II clinical trial. *Int J Radiat Oncol Biol Phys.* Mar 15 2009;73(4):1043-1048.

29. Boike TP, Lotan Y, Cho LC, et al. Phase I dose-escalation study of stereotactic body radiation therapy for low- and intermediate-risk prostate cancer. *J Clin Oncol.* May 20 2011;29(15):2020-2026.

30. Timmerman R, McGarry R, Yiannoutsos C, et al. Excessive toxicity when treating central tumors in a phase II study of stereotactic body radiation therapy for medically inoperable early-stage lung cancer. *J Clin Oncol.* Oct 20 2006;24(30):4833-4839.

31. Tomifuji M, Araki K, Niwa K, et al. Comparison of Voice Quality after Laser Cordectomy with That after Radiotherapy or Chemoradiotherapy for Early Glottic Carcinoma. *ORL; journal for oto-rhino-laryngology and its related specialties.* Mar 20 2013;75(1):18-26.

32. Christiansen H, Rodel RM. [Subjective evaluation of voice quality after surgical or radiation treatment of T1 glottic carcinoma.]. *Strahlentherapie und Onkologie : Organ der Deutschen Rontgengesellschaft ... [et al].* Feb 9 2012.

33. Chu PY, Hsu YB, Lee TL, Fu S, Wang LM, Kao YC. Longitudinal analysis of voice quality in patients with early glottic cancer after transoral laser microsurgery. *Head & neck.* Sep 2012;34(9):1294-1298.

34. Lester SE, Rigby MH, MacLean M, Taylor SM. 'How does that sound?': objective and subjective voice outcomes following CO(2) laser resection for early glottic cancer. *The Journal of laryngology and otology.* Dec 2011;125(12):1251-1255.

35. Jotic A, Stankovic P, Jesic S, Milovanovic J, Stojanovic M, Djukic V. Voice quality after treatment of early glottic carcinoma. *Journal of voice : official journal of the Voice Foundation.* May 2012;26(3):381-389.

36. Rosen CA, Lee AS, Osborne J, Zullo T, Murry T. Development and validation of the voice handicap index-10. *The Laryngoscope.* Sep 2004;114(9):1549-1556.

37. Chen AY, Frankowski R, Bishop-Leone J, et al. The development and validation of a dysphagia-specific quality-of-life questionnaire for patients with head and neck cancer: the M. D. Anderson dysphagia inventory. *Archives of otolaryngology--head & neck surgery.* Jul 2001;127(7):870-876.

38. Spielmann PM, Majumdar S, Morton RP. Quality of life and functional outcomes in the management of early glottic carcinoma: a systematic review of studies comparing radiotherapy and transoral laser microsurgery. *Clinical otolaryngology : official journal of ENT-UK ; official journal of Netherlands Society for Oto-Rhino-Laryngology & Cervico-Facial Surgery.* Oct 2010;35(5):373-382.

39. Bahannan AA, Zabrodsky M, Cerny L, Chovanec M, Lohynska R. Quality of life following endoscopic resection or radio-therapy for early glottic cancer. *Saudi medical journal.* Apr 2007;28(4):598-602.

40. Goor KM, Peeters AJ, Mahieu HF, et al. Cordectomy by CO2 laser or radiotherapy for small T1a glottic carcinomas: costs, local control, survival, quality of life, and voice quality. *Head & neck.* Feb 2007;29(2):128-136.

41. Stoeckli SJ, Schnieper I, Huguenin P, Schmid S. Early glottic carcinoma: treatment according patient's preference? *Head & neck.* Dec 2003;25(12):1051-1056.

42. Lin DY, Feuer EJ, Etzioni R, Wax Y. Estimating medical costs from incomplete follow-up data. *Biometrics.* Jun 1997;53(2):419-434.

43. Tsiatis AA. Estimating the distribution of quality-adjusted life with censored data. *American heart journal.* Apr 2000;139(4):S177-181.

44. Stoeckli SJ, Guidicelli M, Schneider A, Huber A, Schmid S. Quality of life after treatment for early laryngeal carcinoma. *Eur Arch Otorhinolaryngol.* Feb 2001;258(2):96-99.

45. Longo CJ, Bereza BG. A comparative analysis of monthly out-of-pocket costs for patients with breast cancer as compared with other common cancers in Ontario, Canada. *Curr Oncol.* Jan 2011;18(1):e1-8.

46. Zhao H, Tian L. On estimating medical cost and incremental cost-effectiveness ratios with censored data. *Biometrics.* Dec 2001;57(4):1002-1008.

47. Gelber RD, Gelman RS, Goldhirsch A. A quality-of-life-oriented endpoint for comparing therapies. *Biometrics.* Sep 1989;45(3):781-795.
